# Economic and financial crisis based on Troika’s intervention and potentially avoidable hospitalizations: an ecological study in Portugal

**DOI:** 10.1186/s12913-021-06475-4

**Published:** 2021-05-26

**Authors:** Cristina Loureiro da Silva, João Victor Rocha, Rui Santana

**Affiliations:** 1 NOVA National School of Public Health, Universidade NOVA de Lisboa, Av. Padre Cruz, 1600-560 Lisbon, Portugal; 2 Comprehensive Health Research Center, Universidade NOVA de Lisboa, Lisbon, Portugal

**Keywords:** Ambulatory care sensitive conditions, Potentially preventable admissions, Economic and financial crisis, Troika’s intervention

## Abstract

**Background:**

Hospitalisations for Ambulatory Care Sensitive Conditions (ACSC) cause harm to users and to health systems, as these events are potentially avoidable. In 2009, Portugal was hit by an economic and financial crisis and in 2011 it resorted to foreign assistance (“Memorandum of Understanding” (2011–2014)). The aim of this study was to analyse the association between the Troika intervention and hospitalisations for ACSC.

**Methods:**

We analysed inpatient data of all public NHS hospitals of mainland Portugal from 2007 to 2016, and identified hospitalisations for ACSC (pneumonia, chronic obstructive pulmonary disease, hearth failure, hypertensive heart disease, urinary tract infections, diabetes), according to the AHRQ methodology. Rates of hospitalisations for ACSC, the rate of enrollment in the employment center and average monthly earnings were compared among the pre-crisis, crisis and post-crisis periods to see if there were differences. A Spearman’s correlation between socioeconomic variables and hospitalisations was performed.

**Results:**

Among 8,160,762 admissions, 892,759 (10.94%) were classified as ACSC hospitalizations, for which 40% corresponded to pneumonia. The rates of total hospitalisations and hospitalisations for ACSC increased between 2007 and 2016, with the central and northern regions of the country presenting the highest rates. No correlations between socioeconomic variables and hospitalisation rates were found.

**Conclusions:**

During the period of economic and financial crisis based on Troika’s intervention, there was an increase in potentially preventable hospitalisations in Portugal, with disparities between the municipalities. The high use of resources from ACSC hospitalisations and the consequences of the measures taken during the crisis are factors that health management must take into account.

## Background

Hospitalisations for Ambulatory Care Sensitive Conditions (ACSC) are a source of concern for health policy makers, managers and researchers, not only because of their associated high cost but also because of the limitations they create in health management and in the quality of life of the patients [1, 2]. These hospitalisations are considered potentially avoidable, because the hospital admission could have been prevented through timely, effective, continuous and accessible outpatient health care [3]. However, it is important to take into account that these events are influenced by factors within health care (health promotion, chronic disease management, availability of health care providers) and beyond it, such as the health literacy of users, capacity of disease self-management, socioeconomic characteristics and distance to care, to name a few [4–6]. The analysis of hospitalisations for ACSC can help to understand which areas to focus on (health policies, health care, or social sector), even simultaneously [6, 7]. In a geographic analysis, it is possible to see which regions should be prioritised, what needs to be improved (access, prevention, health promotion, diagnosis, treatment, health literacy, socioeconomic status), and which pathologies to focus on [3, 8, 9].

Hospitalisations for ACSC account for a significant share of the total hospitalisations in many countries [1, 3, 5, 8, 10]. In Portugal, it was estimated that around 10.4% of all hospitalisations per year between 2000 and 2014 were for ACSC [4], according to the methodology of the Agency for Healthcare Research and Quality (AHRQ) [11]. According to the methodology by Caminal [12], five conditions made up almost 80% of all hospitalisations for ACSC in Portugal between 2002 and 2013: pneumonia (23.9%), chronic obstructive pulmonary disease (COPD) (14.4%), heart failure (HF) (14.2%), hypertensive heart disease (HD) (14, 2%), and urinary tract infection (UTI) (12.4%) [13]. Diabetes, despite being responsible for only 4% of all hospitalisations for ACSC, is a common comorbidity in many cases [13]. Regarding the geographic distribution of these hospitalisations in mainland Portugal,studies have shown that coastal areas next to large cities (Porto, Coimbra, and Lisbon) had lower rates of hospitalisation for ACSC (the locations near Lisbon have low rates of these hospitalisations; however, the city of Lisbon specifically has the highest rates), in contrast to the interior areas of the centre and north that presented higher rates [3, 8, 13].

Health systems and their features (including hospital care) vary depending on the historic moment the country is experiencing [14]; therefore specific events may be accompanied by variations in the hospitalisations. In 2009, Portugal (as many other countries in the world) was affected by a serious economic and financial crisis that had started in 2007 in the United States of America, in which the Gross Domestic Product (GDP) suffered negative growth in two consecutive quarters, accompanied by an increase in the unemployment rate [15]. Thus, in 2011, the country required external assistance, and a “Memorandum of Understanding” was signed in the same year with the European triumvirate (constituted by the European Commission [EC], International Monetary Fund [IMF] and European Central Bank [ECB], the so-called Troika), which only ended in 2014 [16, 17].

During the intervention of Troika, several measures were implemented to reduce public expenditure (aiming at improving efficiency and effectiveness), which also affected the health sector, which may have had an impact on hospitalisations due to ACSC as well as the respective associated factors [15, 16, 18]. Contrary to what has been done in Portugal, when these types of measures are implemented, which can directly or indirectly affect the population, studies should be carried out prior to the implementation of these measures, as well as monitoring studies, so that their impact is predicted and monitored [19].

In 2011, one of the measures taken was a budget cut of the National Health Service (NHS), in which it was intended that efficiency would increase by decreasing the prices paid to hospitals. However, this increase in efficiency was also accompanied by what is conceptualised as underfunding in health [15, 16]. In addition, in 2012, the rules for attributing exemptions were revised and the co-payment for consultations and emergencies more than doubled (an appointment in primary health care went from 2.25 € to 5.00 €, urgent care from 3.80 € to 10.00 €, and emergency consultation in hospitals from 9.60 € to 20 €) [16, 17, 20]. These co-payments are the same in all municipalities. Other measures taken to reduce expenditure included decreased support for the transportation of patients, reduction in the price of medicines most used by the Portuguese population, implementation of a centralised system of purchases and medication supplies, and reduction of the price paid by the NHS to private institutions with complementary means of diagnosis and treatments [16, 18]. While these changes were occurring in health services delivery, the average income of Portuguese families was decreasing, which led to reduced capacity in purchasing medicines, avoiding seeking health care in justified occasions, and absences from appointments due to the impossibility of missing a day of work or being unable to pay for the consultation [16–18]. Furthermore, people would end up receiving medical care in more advanced states of their diseases, thus generating more costs and lost efficiency [20]. In addition to the decrease in average income, there was also an increase in unemployment rates during this period (from 2008 to 2012, the unemployment rate doubled, from 7.7 to 15.9%, reaching 16.7% in 2013), which is why it is often used as a proxy for an economic crisis (the unemployment rate is the variable that changes most rapidly during an economic and financial crisis) [15, 18]. Perelman et al. found that hospital admissions in Portugal were positively and significantly correlated with the unemployment rate, and the average delay was negatively correlated with that same rate [15].

The impact of changes that occurred during the period of economic and financial crisis has been poorly studied, with focus mostly on the impact that these types of periods have on people’s mental health and on the self-care that they exhibit during such periods [18]. Little has been studied about the increased rates of hospitalisations and hospitalisations for ACSC caused by the increase in moderating fees, restrictions in transport of patients and the increase in the unemployment rate [18]. During the crisis period experienced by the Portuguese population, financial barriers were observed that prevented users from buying medicines necessary to control their chronic pathologies (e.g., COPD, hypertension, HF) and limited access to follow-up treatments [19]. Such situations could have led to hospitalisations that could have been avoided if the patients had been previously treated, with better monitoring and greater frequency [19]. In other countries, such as Spain and Greece, patients with HF and/or hypertensive HD experienced decompensation during the crisis due to lack of self-management and access to health care, generating an increase in admissions and readmissions due to acute myocardial infarction [19]. Also in Greece, there were increases in the unemployment rates and a decrease in income-generated barriers in access to health care [21], similar to what was experienced in Portugal.

Studies regarding health outcomes in periods of economic crisis consider that people’s health deteriorates due to financial constraints and decreased self-care [15, 20]. Therefore, it is plausible to assume that pathologies considered ACSC end up being less controlled, leading to an increase in hospitalisations that could have been avoided. Monitoring of the consequences generated by a crisis is necessary to assist the decision-making process within the scope of health management. Given the representativeness that hospitalisations for ACSC have in the total hospitalisations in Portugal and the high cost they represent, the aim of this study is to test the association between the economic and financial crisis and hospitalisations for ACSC in mainland Portugal, between 2007 and 2016.

## Methods

### Study design and data sources

We conducted an ecological retrospective study. A group of individuals was analysed taking into account geographical (municipality) and temporal (years 2007 to 2016) factors. Through this type of study, new knowledge is created on a topic that has not yet been analysed. In addition, new questions and hypotheses are created that can be analysed through other studies.

We used data referring to hospitalisations from 2007 to 2016 in mainland Portugal (*n* = 8,169,762 hospital admissions), obtained from the hospitalisations database provided by the Portuguese Central Administration of the Health System [*Administração Central do Sistema de Saúde- ACSS*]. This database contains anonymised information such as age, sex, and area of residence of the patient; primary and secondary diagnoses (according to the 9th and 10th revision of the International Classification of Diseases [ICD]); length of stay; and discharge disposition, among other data that were not used in the present study. For data on resident population and average monthly earnings (AME), the source was the Statistics Portugal (SP) database. For unemployment rates, the number of people enrolled in employment centres by municipality was used as a proxy (as information on unemployment per municipality is not regularly collected, only in census years); this information was obtained from the Employment and Vocational Training Institute [*Instituto de Emprego e Formação Profissional- IEFP*].

The analysis for this study included hospitalisations identified as one of the following ACSC: pneumonia, COPD, HF, HD, UTI and diabetes (these were responsible for the majority of hospitalisations due to ACSC in Portugal, according to the study carried out by the World Health Organization [WHO]) [13]. The definition of which hospitalisations were ACSC was made using the AHRQ methodology [22], which identifies prevention quality indicators (PQIs) according to the ICD codes of the main and secondary diagnoses. This methodology did not include hospitalisations of patients aged less than 18 years old, admitted for obstetric admissions, transferred from other health care facilities, or with missing information for age, gender, municipality of residency, and diagnoses codes. Details on inclusion and exclusion criteria and the ICD codes used for the construction of PQIs can be found in the AHRQ guidelines [22].

### Variables and statistical analysis

As a dependent variable, the rate of potentially avoidable hospitalisations per 100,000 adults was used. The hospitalisations were characterised according to the year of admission, municipality of residence of the patient to which they corresponded, age, sex, and ACSC that caused hospitalisation. The ACSC selected corresponded to the PQI 5 (COPD or asthma in elderly adults), 7 (HD), 8 (HF), 11 (pneumonia) and 12 (UTI). For hospitalisations related to diabetes, the following PQIs were used: 1 (short-term complication of diabetes), 3 (long-term complication of diabetes), 14 (uncontrolled diabetes), 16 (lower extremity amputation in patients with diabetes) [22]. As explanatory variables, the employment centre enrollment rate (ECER) per 100 inhabitants of working age (20 to 65 years old) was used (as a proxy for unemployment), as well as the AME. The unit of analysis for the dependent and explanatory variables was the municipality, which numbered 278 for mainland Portugal. More specific information about the municipalities was not included because not much information was available. If the distance to health services or the income of each family was included (information that is not available), the study was no longer ecological.

For the descriptive analysis, first the ACSC were described in terms of absolute numbers, rates, how much they represented for all hospitalisations, the analysis per ACSC, and evolution from 2007 to 2016. To further investigate the evolution of hospitalisation rates across the country, the volume of hospitalisations for ACSC in the years 2007, 2011, and 2016 was analysed, using quintiles, according to the rates for the year 2007, and values presented in maps of Portugal. Then, hospitalisations for ACSC in general were analysed according to the age and age group of the patient (categorised as adult [under 65 years] and elderly [65 years or older]).

To analyse possible associations between the economic and financial crisis and hospitalisations for ACSC in mainland Portugal between 2007 and 2016, first the years of analysis were aggregated in three different groups - 1) pre-crisis (from 2007 to 2010), 2) crisis (from 2011 to 2014), and 3) post-crisis (from 2015 to 2016) - which references the beginning and the end of the Troika’s intervention in Portugal. The average rate of hospitalisations was analysed according to the before/after methodology. That is, through samples, the values ​​of the pre-crisis period were compared with those of the crisis; the values ​​of the crisis period with those of the post-crisis period; and the values ​​of the pre-crisis period with those of the post-crisis period. Subsequently, the normality of the dependent variables (mean of the hospitalisation rates of each ACSC, for the pre-crisis, crisis, and post-crisis periods) was analysed and, as they all followed a non-normal distribution (*p* < 0.05) according to the Shapiro-Wilk and Kolmogorov-Smirnov tests, the Wilcoxon-Mann-Whitney test (non-parametric test) was applied to analyse the difference in means.

To understand if there was any correlation between the socioeconomic variables (rate of enrolment in the employment centre and AME) and the rate of hospitalisations for all causes and ACSC, the average of each of the variables per period was determined. Subsequently, the percentage variation of the averages between the three periods was calculated so that it was possible to compare them. Finally, Spearman’s bivariate correlation between variations in hospitalisation rates and variations in socioeconomic variables was performed. A 5% significance level was adopted in this study. Statistical analysis were performed using the IBM SPSS Statistics 26®, and maps were generated using QGis 3.14®.

## Results

Between 2007 and 2016, out of 8,160,762 hospitalisation incidents registered, 892,759 incidents (10.94%) corresponded to hospitalisations caused by the six ACSC included in the study. Pneumonia is the ACSC with the greatest impact, corresponding to 329,490 hospitalisations (36.91%), followed by 200,027 for HF (22.41%), 142,857 for UTI (16.00%), 102,521 for CPOD (11.48%), 99,935 for diabetes (11.19%), and finally, 17,929 for HD (2.01%).

Table 1 shows that, during the period of analysis there was an increase in the total number of hospitalisations. Regarding hospitalisations for ACSC, there was also an increase. The year with the lowest volume was 2007, and the one with the highest volume was 2015. The share ACSC represented in total hospitalisations increased until 2012, with decreases in 2013/14 and in 2016. The rate of hospitalisations for ACSC per 100,000 adult inhabitants increased during the analysed period, going from 956.50 in 2007 to 1219.17 in 2015. The rates for pneumonia, HF, and UTI increased between 2007 to 2016, but decreased for diabetes. The rates for COPD and HD did not vary greatly over the years.

**Table 1:** Evolution of total hospitalisations, hospitalisations for ACSC and rates, 2007 to 2016, in Portugal and rates of hospitalisation per 100,000 inhabitants per ACSC, 2007 to 2016, Portugal

Year	Population (> 20 years)	Total hospital admissions	Hospital admissions for ACSC	Hospital admissions for ACSC / total hospital admissions (%)	ACSC rates/ 100,000 habitants	Pneumonia/ 100,000 habitants	DPOC/ 100,000 habitants	HF/ 100,000 habitants	HD/ 100,000 habitants	Diabetes/ 100,000 habitants	UTI/ 100,000 habitants
2007	7,948,571	759,345	76,028	10.01	956.50	343.46	127.97	208.45	18.24	147.47	110.90
2008	7,970,100	787,868	79,357	10.07	995.68	351.55	128.02	219.13	20.34	146.95	129.70
2009	7,994,797	783,484	87,433	11.16	1093.62	422.99	130.22	228.32	20.50	142.31	149.28
2010	8,020,482	781,218	88,680	11.35	1105.67	412.66	128.65	233.75	21.72	134.36	174.54
2011	8,020,859	770,296	87,812	11.40	1094.80	418.41	124.95	229.21	23.19	124.38	174.66
2012	7,994,433	801,369	92,765	11.58	1160.37	433.25	138.52	258.52	23.22	123.89	182.98
2013	7,965,403	872,538	95,584	10.95	1199.99	432.51	130.65	274.74	26.56	125.77	209.76
2014	7,940,447	865,473	94,883	10.96	1194.93	430.11	129.59	280.05	24.24	112.79	218.15
2015	7,928,764	868,234	96,665	11.13	1219.17	448.03	132.04	286.97	23.91	104.93	223.29
2016	7,917,454	870,937	93,552	10.74	1181.59	441.23	115.63	291.32	23.04	90.61	219.77

**Fig. 1:**
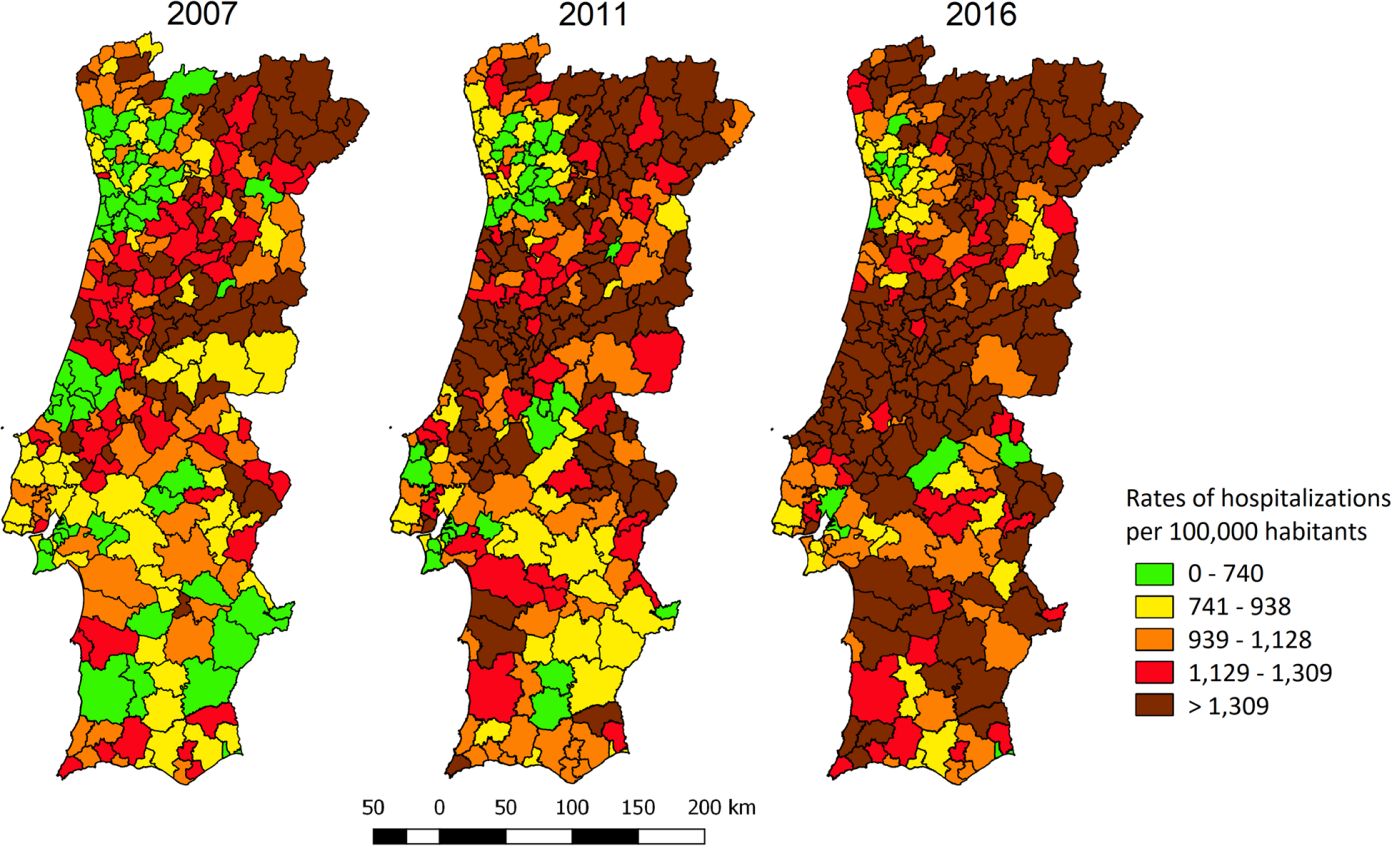
Evolution of rates of hospitalizations for ACSC per 100,000 adult inhabitants, 2007, 2011 and 2016, Portugal [Source: Elaborated by the authors]

In Fig. 1, municipalities were classified according to quartiles of rates of hospitalisation for ACSC per 100,000 inhabitants in 2007. The map shows that, for the 278 municipalities of mainland Portugal, there were 55 with rates higher than 1300 in 2007. This number increased considerably, with 140 municipalities with such values in 2016, with a notable increase in the central and northern regions of the country.

Regarding the age of the patients included in the study, in 2016, only 25% were below the age of 70 years, with an increase of 3.6 years (72.63 to 76.20 years) in the average age of the hospitalised patients due to ACSC between 2007 and 2016. The distribution of hospitalisations by sex is quite similar over the 10 years analysed (about 49% of men and 51% of women) (Table 2).

**Table 2:** Distribution of hospitalizations by ACSC, sex and age category, 2007 to 2016

		Year									
		2007	2008	2009	2010	2011	2012	2013	2014	2015	2016
Age	Q1	66	67	66	68	67	69	69	69	69	70
	Q2	77	77	77	78	78	79	79	79	80	80
	Q3	83	84	84	85	85	85	85	86	86	86
	Average rate	72.63	73.31	73.19	74.05	74.04	75	75.33	75.25	75.84	76.20
	SD	15.33	15.08	15.31	15.04	15.12	14.56	14.54	14.83	14.54	14.50
Sex	Men	49.38%	49.24%	49.48%	49.25%	49.13%	49.16%	48.65%	48.04%	46.86%	48.52%
	Women	50.62%	50.76%	50.52%	50.75%	50.87%	50.84%	51.35%	51.69%	52.14%	51.48%

**Fig. 2:**
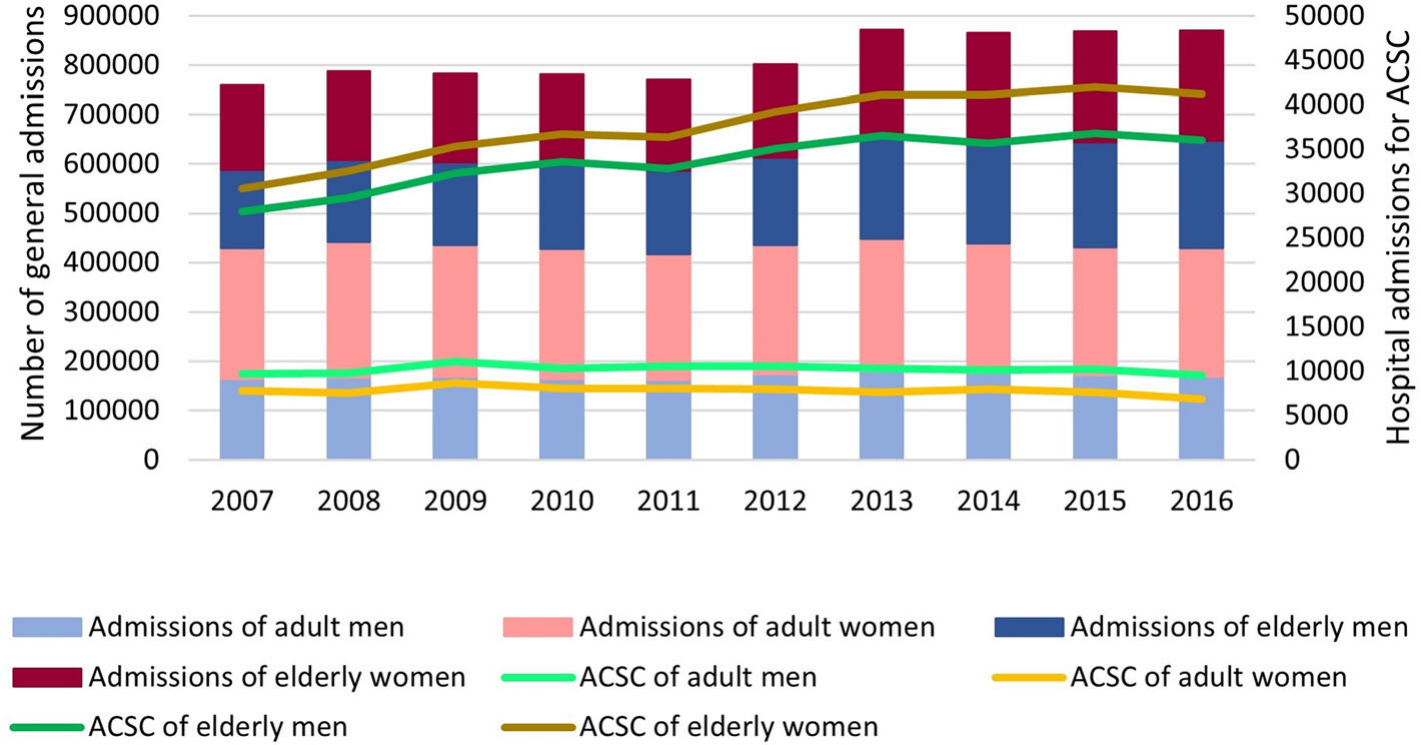
General hospitalizations and hospitalizations for ACSC, by sex and age category, between 2007 and 2016

According to Fig. 2, it is possible to conclude that the volume of hospitalisations relative to adults (ages below 65 years) did not vary considerably; however, it did among the elderly (age equal to or above 65 years). Elderly women were responsible for more hospitalisations for ACSC, and in this case there was also a greater increase in the total volume of hospitalisations.

Using the Wilcoxon statistical test, the rates of hospitalisations for ACSC of the three periods (pre-crisis, crisis, and post-crisis) were compared to analyse if there were significant changes (Table 3). For rates of total hospitalisations and hospitalisations for ACSC, we observed statistically significant increases between the three periods considered (*p* < 0.001). For pneumonia, HF, and UTI, we also observed higher hospitalisation rates in the post-crisis period than those found in the crisis period and in the pre-crisis period, indicating a statistically significant increase (*p* < 0.001). In relation to HD, comparing hospitalisation rates in the crisis and post-crisis periods, and also the pre-crisis period with the post-crisis period, there were significant increases (*p* < 0.01); however, the increase between the crisis and post-crisis periods could not be considered significant (*p* = 0.091). Diabetes was the only ACSC included in the study in which there was a decrease in the rate of hospitalisations over the periods (*p <* 0.001). In the case of COPD, no statistically significant differences were found (*p* > 0.05).

**Table 3:** Comparison of average of hospitalisation rates by periods and correlation between hospitalisation rates and socioeconomic variables, Portugal

Conditions	Periods	Average rate (Standard deviation)	(Minimum, Maximum)	Periods compared	Wilcoxon test (*p-*value)	Variation	Spearman’s correlation E.C.E.R.	Spearman’s correlation A.M.E.
Pneumonia	Pre-crisis	463.96 (186.78)	(142.99; 1232.63)	Pre-crisis / Crisis	Z = −7.590, *p* < 0.001	Increase *	rho = −0.063 *p* = 0.298**	rho = − 0.021 *p* = 0.730**
	Crisis	526.47 (233.01)	(183.17; 1642.86)	Crisis / Post-crisis	Z = −5.061, *p* < 0.001		rho = − 0.005 *p* = 0.940**	rho = 0.137 *p* = 0.022**
	Post-crisis	564.20 (244.00)	(169.41; 2024.63)	Pre-crisis / Post-crisis	Z = − 8.692, *p* < 0.001		rho = − 0.034 *p* = 0.569**	rho = 0.045 *p* = 0.454**
COPD	Pre-crisis	130.72 (62.81)	(25.57; 394.33)	Pre-crisis / Crisis	Z = − 1.382, *p* = 0.167	No difference **	rho = 0.074 *p* = 0.222**	rho = 0.067 *p* = 0.264**
	Crisis	136.09 (77.28)	(9.69; 472.42)	Crisis / Post-crisis	Z = − 1.186, *p* = 0.236		rho = 0.049 *p* = 0.413**	rho = 0.130 *p* = 0.031**
	Post-crisis	135.15 (90.28)	(17.72; 661.67)	Pre-crisis / Post-crisis	Z = − 0.102, *p* = 0.919		rho = − 0.006 *p* = 0.918**	rho = 0.117 *p* = 0.051**
HD	Pre-crisis	23.34 (19.70)	(0.00; 151.82)	Pre-crisis / Crisis	Z = − 4.766, *p* < 0.001	Increase **	rho = − 0.062 *p* = 0.308**	rho = 0.046 *p* = 0.452**
	Crisis	27.56 (23.40)	(0.00; 237.32)	Crisis / Post-crisis	Z = − 1.689, *p* = 0.091		rho = 0.055 *p* = 0.359**	rho = − 0.060 *p* = 0.292**
	Post-crisis	30.31 (28.84)	(0.00; 254.67)	Pre-crisis / Post-crisis	Z = − 4.426, *p* < 0.001		rho = 0.037 *p* = 0.537**	rho = 0.029 *p* = 0.635**
HF	Pre-crisis	253.21 (105.95)	(92.43; 844.69)	Pre-crisis / Crisis	Z = − 6.744, *p* < 0.001	Increase *	rho = 0.043 *p* = 0.480**	rho = 0.103 *p* = 0.087**
	Crisis	282.66 (103.89)	(105.62; 758.37)	Crisis / Post-crisis	Z = − 8.188, *p* < 0.001		rho = 0.106 *p* = 0.077**	rho = 0.057 *p* = 0.345**
	Post-crisis	324.92 (128.17)	(143.16; 875.69)	Pre-crisis / Post-crisis	Z = − 9.904, *p* < 0.001		rho = 0.017 *p* = 0.772**	rho = 0.196 p < 0.001*
UTI	Pre-crisis	142.53 (72.76)	(16.39; 496.12)	Pre-crisis / Crisis	Z = − 12.613, *p <* 0.001	Increase *	rho = − 0.084 *p* = 0.160**	rho = 0.069 *p* = 0.248**
	Crisis	211.83 (117.20)	(44.64; 774.22)	Crisis / Post-crisis	Z = − 8.160, *p* < 0.001		rho = − 0.037 *p* = 0.541**	rho = − 0.031 *p* = 0.602**
	Post-crisis	253.77 (154.71)	(36.42; 1299.14)	Pre-crisis / Post-crisis	Z = − 13.248, *p* < 0.001		rho = 0.040 *p* = 0.510**	rho = 0.049 *p* = 0.417**
Diabetes	Pre-crisis	161.97 (77.79)	(51.38; 761.88)	Pre-crisis / Crisis	Z = − 7.527, *p* < 0.001	Decrease *	rho = − 0.042 *p* = 0.484**	rho = 0.067 *p* = 0.267**
	Crisis	138.66 (64.94)	(54.55; 449.33)	Crisis / Post-crisis	Z = − 9.331, *p <* 0.001		rho = − 0.028 *p* = 0.640**	rho = 0.002 *p* = 0.969**
	Post-crisis	112.27 (55.03)	(22.82; 333.63)	Pre-crisis / Post-crisis	Z = − 11.162, *p <* 0.001		rho = 0.016 *p* = 0.795**	rho = 0.024 *p* = 0.694**
Total ACSC	Pre-crisis	1175.73 (399.98)	(436.28; 2956.83)	Pre-crisis / Crisis	Z = − 10.005, *p <* 0.001	Increase *	rho = − 0.052 *p* = 0.384**	rho = 0.089 *p* = 0.138**
	Crisis	1323.28 (454.36)	(559.88; 3322.35)	Crisis / Post-crisis	Z = − 6.532, *p <* 0.001		rho = 0.024 *p =* 0.694**	rho = 0.084 *p* = 0.162**
	Post-crisis	1420.62 (501.97)	(565.35; 4184.24)	Pre-crisis / Post-crisis	Z = − 10.645, *p <* 0.001		rho = 0.010 *p* = 0.868**	rho = 0.131 *p* = 0.029**
Total hospitalisations	Pre-crisis	10,299.57 (1761.93)	(5076.64; 16,935.37)	Pre-crisis / Crisis	Z = − 4.126, *p <* 0.001	Increase *	rho = − 0.006 *p* = 0.914**	rho = − 0.005 *p* = 0.931**
	Crisis	10,624.96 (1721.43)	(7111.26; 17,345.58)	Crisis / Post-crisis	Z = − 6.421, *p <* 0.001		rho = − 0.027 *p* = 0.652**	rho = − 0.042 *p* = 0.490**
	Post-crisis	11,056.07 (1945.00)	(6897.69; 19,993.25)	Pre-crisis / Post-crisis	Z = − 6.064, *p <* 0.001		rho = − 0.032 *p* = 0.596**	rho = − 0.002 *p* = 0.968**

* Difference considered statistically significant with significance value less than 0.001 (*p* < 0.001)

** The difference found was not considered statistically significant since the value of significance proved to be greater than 0.05 (*p* > 0.05)

Spearman correlations were used to test the correlation between variations in hospitalisation rates found in the three periods under analysis, for the total number of hospitalisations, for hospitalisations for ACSC and for hospitalisations for each of the ACSC, and socioeconomic variables included in the study (ECER and AME) (Table 3). It was found that mostly there was no correlation (Spearman rho < 0.1 *p >* 0.05); for example, correlation between the variations between the post-crisis and pre-crisis periods of hospitalisations for ACSC and ECER was *ρ* = 0.01 (*p*-value = 0.868), and AME was Spearman’s *ρ* = 0.131 (*p-*value = 0.029). In cases where a correlation was found, it can be considered as significant, however negligible (*p-*value < 0.05).

## Discussion

The results of this study indicated that potentially avoidable hospitalisations due to ACSC increased during the crisis and post-crisis periods. However, there were no significant associations between these increases and the socioeconomic variables included in the study. Nonetheless, the increase in hospitalisations during this period leads us to believe that the economic and financial crisis experienced by the Portuguese population somehow played an important role in such increases.

The total volume of hospitalisations and the volume of hospitalisations for ACSC experienced increases considered statistically significant over the analysed periods. A previous study found that, although hospitalisations for ACSC also increased between 2002 and 2013, the total volume of hospitalisations did not [13]. The variations in hospitalisations for ACSC were mainly driven by increases in older age groups, as these were higher than experience for the population aged 65 years or less. As in other studies, regardless of the methodology used, there is an increase in hospitalisations for ACSC at older ages [3, 13].

In 2015, hospitalisations for ACSC reached a value of 1200 cases per 100,000 inhabitants, a higher value compared to 2007 (950 cases per 100,000 inhabitants). Such increases are also shown in the maps in Fig. 1: in 2016, there were 85 more municipalities with more than 1300 hospitalisations for ACSC per 100,000 inhabitants, compared to the 2007 map. As found in a study by Rocha et al. [8], the rates of hospitalisations for ACSC in the interior of the north and centre regions of the country have higher values (municipalities represented in brown in Fig. 1), while the large metropolitan areas of Lisbon and Porto have lower rates (except the city of Lisbon itself, which is one of the municipalities with the highest rates of hospitalisations for ACSC). The reasons that may justify this concentration of hospitalisations for ACSC in the interior regions of the country may be: the distance to health care, the lack of doctors, the delay in providing care, the users’ late resort to health care, the lack of preventive and health promotion measures, among others [4, 8, 9, 13, 23]. It would be important to study this information in more detail, as it may reflect some limitation in access or low quality of health care.

Over the 10 years analysed, there were increases in rates of hospitalisations for pneumonia and UTI (acute conditions) and for HD and HF (chronic conditions), and there were statistically significant differences when comparing the pre-crisis, crisis and post-crisis periods. The increase of rates for pneumonia and HF corroborated the results found in a study by the WHO [13]. However, it would be expected that hospitalisation rates for pneumonia, HF, and HD would follow the same trend as observed for diabetes (i.e., would have decreased), since these pathologies are included in the results indicator that is part of the Global Performance Index and, consequently, part of the contractualisation process of the PHC (Primary Health Care) units [13, 24]. In the study by the WHO [13], hospitalisations for COPD experienced significant increases over the period 2002 to 2013; our study did not corroborate such information for the period 2007 to 2016, as there were no significant variations found.

After a major change in a country, as in the case of an economic and financial crisis, in which it is necessary to design and implement strict measures to decrease public expenditure, it is important that studies are carried out to predict and monitor the impact on the population [15–17]. Between May 2011 and May 2014, the “Memorandum of Understanding” prompted the implementation of measures to decrease public expenditure in the health sector as well. However, no study has yet been carried out to ascertain whether these led to decreases in the response capacity of the health system or decreases in the user’s capacity to pay for health needs (e.g., drugs, transport, consultations) [16–20]. Our study intended to fill some of the knowledge gap in this area.

Nevertheless, although there were differences in hospitalisation rates over the analysed periods, when correlating the variations in hospitalisation rates with the variation in ECER and with the AME, no significant correlations were found; when found, these could only be considered negligible. Thus, other factors may be behind this increase in hospitalisations. ACSC hospitalisations are influenced by factors related to the health system directly (health promotion activities, chronic disease management, timely diagnosis and treatment, availability of healthcare providers, clinical indecision), and by factors that are not associated with the referred system (patient education, ability to self-manage the disease itself, social and economic characteristics, advanced age of the user, distance to places of care) [4–6]. In future studies, it would be interesting to see if some of these characteristics have changed before, during, and after the Troika’s intervention and to analyse whether they are related to the increase in hospitalisations. Therefore, it was not possible to corroborate previous findings in the literature that average earnings are inversely associated to rates of hospitalisations for ACSC [25–30].

Between 2007 and 2016, about 1 in every 10 hospitalisations that occurred in public hospitals in mainland Portugal was caused by pathologies that could have been treated or controlled in the outpatient setting. This result corroborates the conclusions of the study by Dimitrovová et al. [4], in which 10.4% of hospitalisations between 2002 and 2013 were for ACSC. Around 75% of all hospitalisations for ACSC occurred in patients aged 70 or over, and the distribution between males and females was quite similar. The ACSC with the greatest impact on the values found were pneumonia (an acute condition, accounting for 36.91% of all hospitalisations for ACSC), followed by HF (a chronic condition, accounting for 22.41%). The remaining four ACSC (UTI, COPD, HD, and diabetes) accounted for the other 40.68% of all hospitalisations for ACSC. As found in Rocha’s study, more than half of hospitalisations resulted from acute illness (about 60%) [8]. In previous studies, pneumonia was also the ACSC with the greatest impact on these hospitalisations [3, 8, 13].

The overall differences in results found between similar studies and ours, mentioned throughout this discussion section, could be due to different methodologies to define ACSC and that the periods of analysis were also different. In a study using the CIHI (Canadian Institute of Health Information) methodology, only 4.5% of hospitalisations in 2012 were caused by ACSC because this methodology only includes chronic diseases and does not include elderly people [1, 3]. In the case of a study carried out with the methodology of Caminal et al., 32.5% of hospitalisations in 2012 were caused by ACSC because this methodology had a greater number of conditions [3, 13]. However, the AHRQ methodology has been widely used and has been subject to timely reviews.

The present study has some limitations. As an ecological study, there is the risk of committing an ecological fallacy; that is, sometimes interpretations for individuals are made from data that refer to group observations [7]. In addition, it was not possible to control confounding variables, which influence the results and are usually unknown to the researcher. In the case of this study, the results were likely more limited due to the difficulty of representing something like the economic and financial crisis through only two variables. Moreover, the variable ECER had to be used as a proxy for unemployment, which inevitably underestimates real unemployment rates, given that many people who are unemployed are not enrolled in employment centres. The AME do not provide information about the income inequalities within the municipalities, as it consists only of an average, but there are no data available to capture these differences. In addition, with regard to obtaining data on hospital admissions generated by ACSC, in most cases the data source is an administrative database [31]. When it comes to obtaining data on the private sector, it is more difficult because the data are not available [31]. Thus, this and other studies do not include data on private hospital admissions because they are not available, but if that information were included, it could change the results of this study. The results found in the study can be understood as an alert for the decision makers in the area of health management, because they could signal problem or failure in the system, and that those could be improved, however further studies will be needed to better understand the results obtained. The hospitalisations for ACSC cause harm to the user and generate high costs for the health system: a hospitalisation has generally a higher cost than care provided in the outpatient setting, and the difference in value could be used to fill other needs of the health system. To reduce this type of hospitalisation may require work between different levels of health care to understand where and how it is necessary to act so the user does not feel the need to resort to emergency services, or that his/her health status did not deteriorate enough to render the hospitalisation needed [2, 13].

This integration of care would allow the user to be placed at the centre of the system and, in this way, create value for the patient [32]. In addition, it is important that when applying more restrictive measures to reduce public expenditure, the impact on the population is monitored. The demand for providing efficient and effective care with less funding is challenging for health professionals, and it may take some time for teams to reorganise themselves, so that the quality of health care is not altered.

## Conclusion

Between 2007 and 2016, there was an increase in hospitalisations for ACSC in mainland Portugal. About 10% of all hospitalisations registered were for ACSC over the analysed period, with pneumonia and HF accounting for the highest proportion. For nearly all conditions, there were increases in hospitalisation rates between the pre-crisis, crisis, and post-crisis periods. Regarding the geographic distribution of these hospitalisations, the interior areas in the north and centre regions showed higher rates. Although no associations were found between these increases and the socioeconomic variables included in the study, it is likely the economic and financial crisis experienced in Portugal played an important role in the significant differences over periods.

More in-depth studies should be carried out to conclude the effective impact of the economic and financial crisis on potentially avoidable hospitalisations. In addition, the differences in rates found between regions of Portugal could mean that there are limitations in access to care and differences in quality in some municipalities, which should be analysed in future studies and addressed by decision-making actors. This is an ecological study, so although concrete conclusions could not be obtained, it created questions that could function as alerts for decision makers and give important information to be further investigated in future studies.

The study contributed to the area of health management, by attributing importance to potentially avoidable hospitalisations due to ACSC, which, in addition to generating high costs for the system and challenging the management of health units, are stressful and harmful for users. Moreover, it emphasises the need and importance of monitoring when restrictive measures are applied, especially in the health area.

## Data Availability

The data of hospitalisations are the property of Central Administration of the Health System (Administração Central do Sistema de Saúde (ACSS), I.P.), however the data are available from the authors request and with permission of the ACSS. The data of hospitalisations are not publicly available, however the authors confirm that interested researchers can ask for access to these data by contacting ACSS directly in the following: Parque da Saúde da Lisboa, Edifício 16, Avenida do Brasil, 53 1700–063 Lisboa, Portugal (e-mail: geral@acss.min-saude.pt).

## Abbreviations

ACSC: Ambulatory care sensitive conditions
ACSS: *Administração Central do Sistema de Saúde* (Portuguese Central Administration of the Health System)
AME: Average monthly earnings
COPD: Chronic obstructive pulmonary disease
EC: European Commission
ECB: European Central Bank
ECER: Employment center enrollment rate
GDP: Gross domestic product
HD: Hypertensive heart disease
HF: Heart failure
ICD: International Classification of Diseases
IEFP: *Instituto de Emprego e Formação Profissional* (Employment and Vocational Training Institute)
IMF: International Monetary Fund
NHS: National Health System
PQIs: Prevention quality indicators
UTI: Urinary tract infection
SP: Statistic Portugal database
